# Low-Level Gestational Lead Exposure Increases Retinal Progenitor Cell Proliferation and Rod Photoreceptor and Bipolar Cell Neurogenesis in Mice

**DOI:** 10.1289/ehp.1002524

**Published:** 2010-09-14

**Authors:** Anand Giddabasappa, W. Ryan Hamilton, Shawntay Chaney, Weimin Xiao, Jerry E. Johnson, Shradha Mukherjee, Donald A. Fox

**Affiliations:** 1 Department of Biology and Biochemistry and; 2 College of Optometry, University of Houston, Houston, Texas, USA; 3 Department of Natural Sciences, University of Houston–Downtown, Houston, Texas, USA; 4 Department of Pharmacology and Pharmaceutical Sciences, University of Houston, Houston, Texas, USA

**Keywords:** bipolar cells, development, gestational exposure, glia, lead, mice, neurogenesis, proliferation, retina, rod photoreceptors

## Abstract

**Background:**

Gestational lead exposure (GLE) produces novel and persistent rod-mediated electroretinographic (ERG) supernormality in children and adult animals.

**Objectives:**

We used our murine GLE model to test the hypothesis that GLE increases the number of neurons in the rod signaling pathway and to determine the cellular mechanisms underlying the phenotype.

**Results:**

Blood lead concentrations ([BPb]) in controls and after low-, moderate-, and high-dose GLE were ≤ 1, ≤ 10, approximately 25, and approximately 40 μg/dL, respectively, at the end of exposure [postnatal day 10 (PND10)]; by PND30 all [BPb] measures were ≤ 1 μg/dL. Epifluorescent, light, and confocal microscopy studies and Western blots demonstrated that late-born rod photoreceptors and rod and cone bipolar cells (BCs), but not Müller glial cells, increased in a nonmonotonic manner by 16–30% in PND60 GLE offspring. Retinal lamination and the rod:cone BC ratio were not altered. *In vivo* BrdU (5-bromo-2-deoxyuridine) pulse-labeling and Ki67 labeling of isolated cells from developing mice showed that GLE increased and prolonged retinal progenitor cell proliferation. TUNEL (terminal deoxynucleotidyl transferase dUTP nick end labeling) and confocal studies revealed that GLE did not alter developmental apoptosis or produce retinal injury. BrdU birth-dating and confocal studies confirmed the selective rod and BC increases and showed that the patterns of neurogenesis and gliogenesis were unaltered by GLE.

**Conclusions:**

Our findings suggest two spatiotemporal components mediated by dysregulation of different extrinsic/intrinsic factors: increased and prolonged cell proliferation and increased neuronal (but not glial) cell fate. These findings have relevance for neurotoxicology, pediatrics, public health, risk assessment, and retinal cell biology because they occurred at clinically relevant [BPb] and correspond with the ERG phenotype.

Perinatal exposure to environmental toxicants such as lead, methylmercury, pesticides, and polychlorinated biphenyls increases the risk of developmental disabilities, mental retardation, neuorosensory alterations, and psychiatric morbidity (Grandjean and Landrigan 2006; Mendola et al. 2002). The spectrum of neurotoxic effects depends on the perinatal period of exposure, concentration and duration of exposure, and genetic susceptibility (Grandjean and Landrigan 2006; Rice and Barone 2000). This is exemplified best by lead exposure, which is especially neurotoxic to the developing central nervous system: Children with blood lead concentrations ([BPb]) < 10 μg/dL—the current low level of concern [Centers for Disease Control and Prevention (CDC) 1991]—have cognitive deficits and neurosensory alterations (Grandjean and Landrigan 2006; Rothenberg et al. 2002).

The phenotype of retinal alterations is markedly dependent on the developmental exposure period. After postnatal lead exposure, rod photoreceptor-selective apoptosis, persistent rod-mediated (scotopic) electroretinographic (ERG) subnormality, and scotopic behavioral deficits occur in humans, monkeys, and rodents (Fox and Boyes 2008; Fox et al. 1997; He et al. 2003). In contrast, children, monkeys, and rats with low-dose (LD) to moderate-dose (MD) gestational lead exposure (GLE) exhibit novel scotopic ERG supernormality (Fox et al. 2008; Lilienthal et al. 1994; Nagpal and Brodie 2009; Rothenberg et al. 2002).

The adult mammalian retina consists of six neuronal cell types and a Müller glial cell (MGC) that originate from a pool of multipotent retinal progenitor cells (RPCs) (Livesey and Cepko 2001; Young 1985a). Retinogenesis proceeds in two distinct yet overlapping histogenetic periods, characterized by the development of early-born cells (ganglion, horizontal, cone, and amacrine cells) mostly during embryogenesis and late-born cells [rods, bipolar cells (BCs), and MGCs] during early postnatal development (Livesey and Cepko 2001; Martins and Pearson 2008; Young 1985b). Approximately 70% of adult [postnatal day 60 (PND60)] mouse retinal cells are rods, and 20% are BCs (Strettoi and Volpini 2002; Young 1985a). Extrinsic factors such as neurotransmitters and modulators alter the cell fate of RPCs; however, these changes are limited by the intrinsic properties of RPCs (Livesey and Cepko 2001; Martins and Pearson 2008). LD and MD GLE produced a novel retinal phenotype in PND60–PND90 rats characterized by an increased number of cells in the outer nuclear layer (ONL) and inner nuclear layer (INL) with no change in glial fibrillary acid protein content, suggesting that GLE selectively increased the number of late-born neurons but not MGCs (Fox et al. 2008).

The goals of our study were to use our mouse model of GLE (Leasure et al. 2008) to test the hypothesis that GLE selectively increases neurons in the scotopic signaling pathway and to determine the cellular mechanisms underlying the phenotype. To accomplish this, we determined the number and distribution of early- and late-born retinal cell types in PND60 control and GLE offspring; kinetics of RPC mitosis, proliferation, and cell-specific apoptosis during development; and spatiotemporal pattern and number of developing late-born cells with 5-bromo-2-deoxyuridine (BrdU) birth dating. The results reveal that GLE increases and prolongs RPC proliferation without decreasing apoptosis. This produced an adult retina with normal lamination and a selectively increased number of rods and BCs.

## Materials and Methods

### Animal model

All experimental and animal care procedures complied with the National Institutes of Health (NIH) Public Health Service Policy on the Humane Care and Use of Laboratory Animals (NIH 2002) and were approved by the Institutional Animal Care and Use Committee of the University of Houston. All animals were treated humanely and with regard for alleviation of suffering. The GLE model, as described by Leasure et al. (2008), uses C57BL/6 mice. Briefly, female mice fed Purina lab chow 5001 (Purina Mills, St. Louis, MO) were given water (control) or water containing a low (27 ppm), moderate (55 ppm), or high (109 ppm) concentration of lead 2 weeks before mating, during pregnancy, and through PND10 to produce a human-equivalent GLE. Dams were mated with a control male overnight, and the presence of a vaginal plug was recorded as gestation day (GD) 0.5. On PND10, we replaced the lead solutions with water. Litter sizes were maintained at six pups each until weaning (PND21). Mice were sacrificed by decapitation between 1000 and 1200 hours on PND1, PND3, PND5, PND7, PND10, and PND60. We observed no differences between sexes for any end point.

As described by Leasure et al. (2008), control, LD, MD, and high-dose (HD) GLE groups had peak [BPb] on PND0 and/or PND10 of 0.72 ± 0.07, 10.10 ± 0.65, 27.23 ± 1.39, and 42.06 ± 0.70 μg/dL, respectively; on GD14 the dam’s [BPb] was similar to that of PND0 pups; and on PND30 the [BPb] in GLE mice were not different from those in controls (*n* = 10–15 mice/treatment group/age). There were no statistical differences between control and GLE groups on any dam measure, litter measure, or body weight.

### Retinal epifluorescent, light, and immunocytochemistry/confocal microscopy studies

All tissue processing, image acquisition, and analysis procedures were as described previously (Fox et al. 1997, 2008; He et al. 2003; Johnson et al. 2007). Briefly, eyes were removed and immersed in ice-cold phosphate-buffered saline (PBS), corneas were slit, and eyes were immersion fixed in buffered 4% paraformaldehyde for 30 min (confocal microscopy) or Karnovsky’s fixative (light microscopy). For confocal microscopy, central sections (10 μm thick) from cryoprotected frozen retinas were cut at 200–400 μm from the optic nerve. Nuclear dyes and primary antibodies directed against retinal-cell-type–specific and cell-cycle markers were used (Table 1). ONL and INL thickness was measured on 4′,6-diamidino-2-phenylindole (DAPI)-stained slides using a calibrated filar micrometer eyepiece. For light microscopy, superior central retinal sections were embedded in Araldite (Electron Microscopy Sciences, Hatfield, PA), sectioned (1 μm), and stained with toluidine blue for rod and cone photoreceptor counting. Twenty fields, each 100 μm in length, were examined.

For all studies, slides (three nonadjacent coded sections) from each treatment group were analyzed using identical exposure and scanning parameters. The images were processed identically using Adobe Photoshop software (Adobe Systems Incorporated, San Jose, CA). All cell counts were conducted using Image-Pro Plus (Media Cybernetics Inc., Bethesda, MD) by an observer who was masked to the exposure type and who used unbiased stereology and morphologic criteria, as described previously (He et al. 2003; Lucocq 2007).

For pulse-labeling cells in S phase (Rachel et al. 2002), BrdU (50 μg/g) was injected intraperitoneally (IP) into pregnant dams on GD16.5 and GD18.5, and pups were injected on PND1, PND3, PND5, and PND7. Mice were sacrificed 2.5 hr after injection. For birth dating late-born retinal cells, two doses of BrdU (100 μg/g) were injected into PND1, PND3, PND5, and PND7 pups separated by 2 hr, and mice were sacrificed on PND60. Slides were triple labeled with an anti-BrdU antibody, the nuclear stain DRAQ5, and antibodies for rods, BCs, or MGCs and then processed for confocal microscopy and counting as previously described (He et al. 2003; Johnson et al. 2007). We used staining, morphological, and pixel density criteria (≥ 20 pixels at 300 dpi) to identify and count BrdU-immunoreactive (BrdU-IR) cells, as previously described (Rachel et al. 2002).

### Western blotting

Immunoblotting was performed as described previously (Fox et al. 2008; He et al. 2003). Briefly, both retinas were removed, cleaned in PBS, and frozen at −80°C until use. Thawed retinas were homogenized in lysis buffer and centrifuged, and 20–30 μg protein was loaded onto SDS-PAGE gels. Blots were probed with selected primary antibodies using GADPH as a loading control (Table 1), incubated with horseradish peroxidase–conjugated secondary antibody, and visualized with ECL Plus (GE Healthcare, Piscataway, NJ). Densitometry measurements were obtained using ImageJ software (NIH 2009) from nonsaturated blots.

### Immunolabeling dissociated progenitor cells

We followed the procedure for single-cell dissociation of PND2, PND4, and PND6 retinas as described previously by He et al. (2000), except that we replaced HEPES with Hank’s balanced salt solution. Dissociated cells (10^6^ cells/mL) were pipetted onto slides, fixed with 4% paraformaldehyde, rinsed, and air dried. Coded slides were double labeled for Ki67 and DRAQ5 and processed for confocal microscopy and counting as previously described (He et al. 2000).

### Terminal deoxynucleotidyl transferase dUTP nick end labeling (TUNEL)

Apoptotic cells were labeled using the ApoAlert DNA Fragmentation Assay TUNEL Kit (Clontech, Mountainview, CA) according to manufacture’s procedure. Briefly, retinal sections were dried and fixed with 4% paraformaldehyde, incubated with proteinase K, and washed, fixed, and washed again. Slides were incubated with equilibrium buffer, sequentially incubated with *terminal* deoxynucleotidyl transferase (TdT) and sodium chloride/sodium citrate, washed, and coverslipped with Vectashield Mounting Medium with DAPI (Vector Laboratories). Positive-control (DNase1) and negative-control (no TdT) slides were processed simultaneously for each experiment. Fluorescent apoptotic cells were identified in different retinal layers using stringent morphologic criteria and counted as previously described (He et al. 2000, 2003).

### Statistical analysis

Only one animal per litter was used for each measure. Data represent four to seven retinas, each from a different mouse, at each age per treatment group. Group data were analyzed by analysis of variance followed by post hoc multiple comparisons using Tukey’s honestly significant difference test (KaleidaGraph; Synergy Software, Reading, PA). Data are presented as mean ± SE, and the difference from controls was regarded as significant if *p* < 0.05.

## Results

### GLE increased retinal ONL and INL thickness

**Figure 1:**
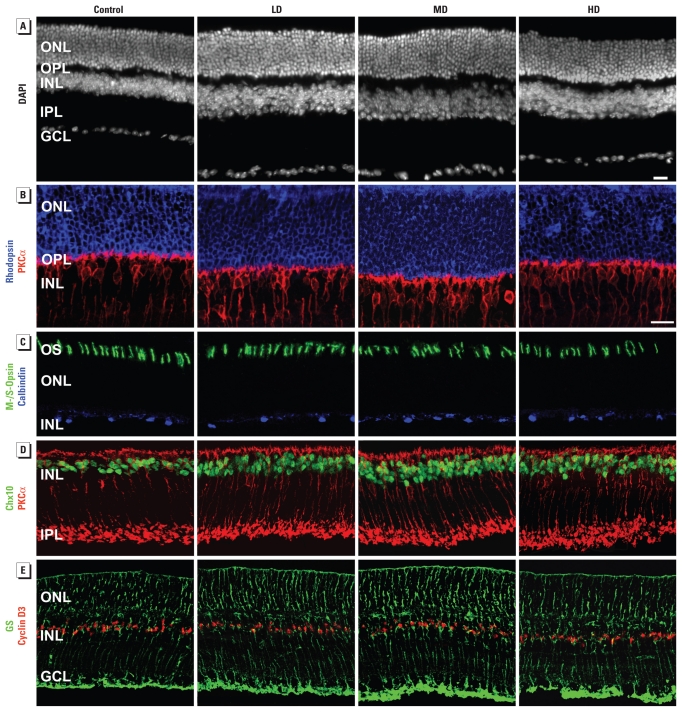
GLE selectively increases the number of rod photoreceptors and BCs in the adult (PND60) mouse retina as shown in light and confocal microscopy studies. (*A*) Representative DAPI nuclear labeling shows that ONL (rod and cone nuclei), INL (horizontal, bipolar, amacrine, and MGC nuclei), outer plexiform layer (OPL), inner plexiform layer (IPL), and total retinal thickness increased in retinas from animals in the LD, MD, and HD GLE groups. Retinal GCL cellularity and retinal lamination were not different in control and GLE retinas. (*B*–*E*) Representative double-labeled confocal microscopy studies reveal rod and BC selectivity of the retinal phenotype. (*B*) The number of rhodopsin-IR rod nuclei and PKCα-IR rod BC somas—in distal (upper) INL—increased in GLE retinas. (*C*) The numbers of cone outer segments (OS) immunoreactive for middle- and short-wavelength–sensitive opsin (M-/S-opsin) and horizontal cells immunoreactive for calbindin were not different in control and GLE retinas. (*D*) The numbers of Chx10-IR BC nuclei and Chx10/PKCα colabeled rod BCs increased in adult GLE retinas. (*E*) The number of cyclin D3–IR MGCs colabeled with glutamine synthetase (GS), were similar in adult control and GLE retinas. Bars = 20 μm for *A* and for *B–E*.

**Figure 2:**
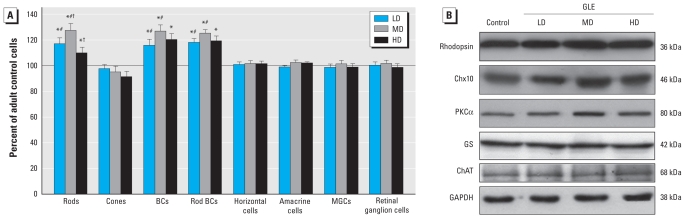
GLE selectively increases the number of rod photoreceptors and BCs in retinas from adult mice. (*A*) Unbiased stereological analyses of major retinal cell types in adult retinas show that LD, MD, and HD GLE produced selective and significant nonmonotonic increases in the number of rods and BCs, relative to controls. Values are mean ± SE from three nonadjacent sections per retina from four to seven retinas per treatment group, with each retina from a different mouse. (*B*) Representative Western blots reveal that GLE increased the retinal content of rhodopsin, Chx10, and PKCα but did not change the amount of glutamine synthetase (GS) or amacrine cell ChAT content. GAPDH was used as the protein loading control. *p < 0.05, compared with control. Groups sharing # or † were significantly different within the cell type at *p* < 0.05.

DAPI nuclear staining revealed that GLE increased the cell density and thickness of the ONL and INL in PND60 mice (Figure 1A). GLE also increased the thickness of the outer and inner plexiform (synaptic) layers and total retinal thickness (Figure 1A). The central ONL and INL of controls contained 10–11 and 4–5 nuclei/layer, respectively, and were 54.0 ± 2.4 and 37.2 ± 2.1 μm thick (mean ± SE), respectively. In LD, MD, and HD GLE mice, ONL thickness significantly increased by 18.3 ± 1.5%, 25.5 ± 2.2%, and 8.4 ± 1.2%, respectively; INL thickness significantly increased by 21.4 ± 1.6%, 29.8 ± 2.5%, and 18.0 ± 1.9%, respectively; and total retinal thickness significantly increased by 25.7 ± 2.1%, 30.4 ± 2.8%, and 15.7 ± 1.5%, respectively. The number of DAPI-stained nuclei in the retinal ganglion cell layer (GCL) of controls (15.4 ± 1.5 per 100 μm length) was not significantly different in GLE mice (Figures 1A and 2A).

### GLE selectively increased the number of rods

We conducted additional studies to characterize and quantify the cell-specific increase in ONL thickness. Rhodopsin-labeled retinas showed that GLE increased ONL cellularity and thickness (Figure 1B). In GLE mice, the density of cone outer segments immunoreactive for middle- and short-wavelength–sensitive opsin was not significantly different from controls (Figure 1C; 15.4 ± 0.8 per 100 μm length). Stereologic analysis showed that GLE selectively and significantly increased the number of rods (Figure 2A). In controls, the numbers of central rod and cone nuclei were 126.5 ± 3.4 and 3.9 ± 0.2 per 100 μm length, respectively, consistent with previously published results (He et al. 2003). In LD, MD, and HD GLE mice, the numbers of rods significantly increased by 16.9 ± 4.7%, 27.3 ± 5.3%, and 9.5 ± 1.6%, respectively; however, cone density was unchanged (Figure 2A). Immunoblots showed that the rhodopsin content significantly increased in LD, MD, and HD GLE retinas by 21.1 ± 1.5%, 34.3 ± 2.7%, and 14.0 ± 2.6%, respectively (Figure 2B). Collectively, these findings demonstrate that GLE increased the number of rod, but not cone, photoreceptors in PND60 mice. Furthermore, these nonmonotonic effects for the stereology and Westerns blots were characterized by an inverted U-shaped dose–response curve.

### GLE selectively increased the number of BCs

Additional studies were conducted to determine the INL cell types that increased. We double-labeled retinal sections with an anti-Chx10 antibody, a selective marker for differentiated rod and cone BCs, and an anti-protein kinase Cα (anti-PKCα) antibody that selectively labels rod BCs (Green et al. 2003). In GLE retinas, the number of Chx10-IR BCs increased (Figure 1D). In controls, we observed 56.8 ± 4.5 Chx10-IR cells per 100 μm length of central retina. In LD, MD, and HD GLE mice, this significantly increased by 16.3 ± 4.7%, 26.7 ± 4.7%, and 19.8 ± 5.2%, respectively (Figure 2A). Immunoblots showed that Chx10 content significantly increased in LD, MD, and HD GLE retinas by 18.3 ± 1.6%, 31.4 ± 2.8%, and 20.4 ± 3.1%, respectively (Figure 2B). In GLE retinas, the number of PKCα-IR rod BCs exhibited similar increases (Figure 1D). The number of PKCα-IR rod BCs in control central retina was 18.4 ± 1.5 per 100 μm length, and they significantly increased in LD, MD, and HD GLE mice by 17.9 ± 3.1%, 24.9 ± 3.7%, and 18.9 ± 2.7%, respectively (Figure 2A). This yielded rod BC:total BC ratios of 0.31–0.34 for control and GLE retinas, which are normal ratios for mammalian retina (Strettoi and Volpini 2002). Immunoblots showed that PKCα content significantly increased in LD, MD, and HD GLE retinas by 19.4 ± 1.4%, 24.8 ± 2.2%, and 21.0 ± 2.5%, respectively (Figure 2B). In control and GLE mice, the density of calbindin-IR horizontal cells (Figure 1C) and cyclin D3-IR MGCs (Figure 1E) in central retina (1.4 ± 0.1 and 10.5 ± 0.5 per 100 μm length, respectively) was not significantly different (Figure 2A). Immunoblots for glutamine synthetase revealed no significant differences (Figure 2B), consistent with the confocal and morphometric MGC results (Figures 1E and 2A). We observed no glial fibrillary acid protein labeling or content change in any retinas (Fox DA, Giddabasappa A, Chaney S, unpublished data), similar to our results in GLE rats (Fox et al. 2008), indicating that GLE produced no injury response. The density of choline acetyltransferase-IR (ChAT-IR), γ-aminobutyric acid–IR, and Disabled-1–IR (Dab1 glycinergic AII) amacrine cells in control central INL was 1.9 ± 0.2, 7.3 ± 0.5, and 4.8 ± 0.3 per 100 μm length, respectively. This yielded a Disabled-1:ChAT INL amacrine cell ratio of 2.5:1, which is consistent with our calculated values from published data (Jeon et al. 1998; Rice and Curran 2000). We found no significant differences in these amacrine cell densities in GLE retinas (Figure 2A). Immunoblots for ChAT confirmed the stereology results (Figure 2B). These composite results demonstrate that GLE selectively and proportionately increased the number of rod and cone BCs without altering the cell fate of other cells. Furthermore, the nonmonotonic effects were characterized by an inverted U-shaped dose–response curve.

### BrdU birth-dating studies

**Figure 3:**
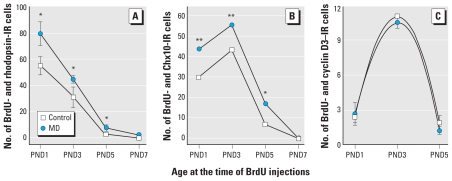
BrdU birth-dating and confocal studies demonstrate that MD GLE selectively and significantly increased the number of rods and BCs in retinas from adult mice. GLE did not alter the kinetics of neurogenesis and gliogenesis. BrdU-IR and rhodopsin-IR rods (*A*) and BrdU-IR and Chx10-IR BCs (*B*) increased in GLE retina, compared with controls. (*C*) No differences in the number of BrdU-IR and cyclin D3–IR MGCs were observed in control and GLE retinas. Values are mean ± SE of IR cells per 400 μm of central retina and and represent from four to seven retinas at each age per treatment group, with each retina from a different mouse. *p < 0.05, and **p < 0.01 compared with controls.

To determine the spatiotemporal kinetics of late-born retinal cells, we conducted BrdU double-labeling experiments with anti-rhodopsin (Figure 3A), anti-Chx10 (Figure 3B), or anti-cyclin D3 antibodies (Figure 3C). GLE selectively and significantly increased rod and BC neurogenesis by 35–40% from PND1 to PND5 while maintaining normal cellular migration and lamination, which confirms and extends our findings. For control and GLE, the rod, BC, and MGC peak birth dates were PND1, PND3, and PND3, respectively, consistent with previously published results (Young 1985b). Thus, MD GLE did not disrupt the initiation or pattern of neurogenesis or gliogenesis, despite increasing rod and BC neurogenesis by approximately 30% (Figure 2A). Two possible, although not mutually exclusive, mechanisms might underlie these selective changes: increased proliferation and/or decreased apoptosis.

### GLE increased and prolonged the proliferation of RPCs

**Figure 4:**
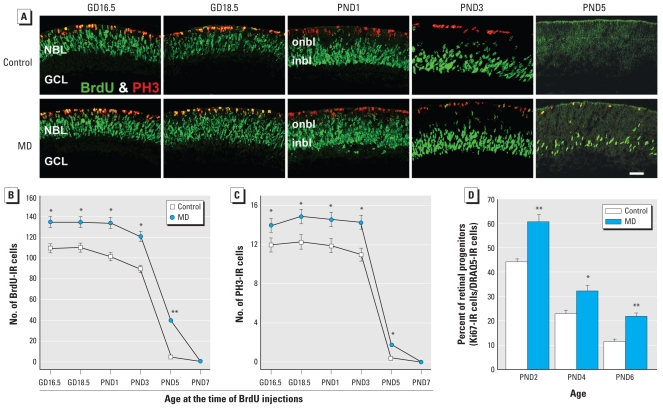
MD GLE increases and prolongs RPC proliferation *in vivo* and *ex vivo.* (*A*) The M-phase marker PH3 and S-phase marker BrdU are present until PND3 in controls and PND5 in GLE central retina. They colocalize in the distal NBL. At all ages, except PND7, there are more BrdU-IR and PH3-IR cells in the GLE retinas. Abbreviations: inbl, inner NBL; onbl, outer NBL. Bar = 20 μm. Stereological analysis of BrdU (*B*) and PH3 (*C*) labeling shows that GLE significantly increased and prolonged RPC proliferation. (*D*) Dissociated single cells from PND2, PND4, and PND6 control and MD GLE retinas were double labeled with an anti-Ki67 antibody and DRAQ5; results reveal that GLE significantly increased RPCs in an age-dependent manner, similar to that shown in *B*. In *B–D*, values are mean ± SE from four to seven retinas at each age per treatment group, with each retina from a different mouse; in *B* and *C,* values represent IR cells per 400 μm of central retina. *p **< 0.**05, and **p < 0.01 compared with control.

We conducted BrdU pulse-labeling studies to determine if the increased rod and BC neurogenesis resulted from increased RPC proliferation. Figure 4A shows that BrdU labeled the central neuroblastic layer (NBL) from GD16.5 to PND3, consistent with the termination of DNA synthesis by PND5 (Young 1985a). The labeling pattern in controls (Figure 4B) was similar to that for [^3^H]-thymidine labeling in developing rat retinal explants (Alexiades and Cepko 1996). In MD GLE retinas, BrdU labeling persisted until PND5 (Figure 4A,B); we observed no BrdU-IR cells on PND7 (Figure 4B). From GD16.5 to PND3, GLE significantly increased the number of BrdU-IR cells by 30% (Figure 4B).

To assess whether GLE affected the spatiotemporal pattern and/or number of RPCs undergoing mitosis, we double-labeled retinas with anti-phosphohistone H3 (anti-PH3) and anti-BrdU antibodies. PH3 labeled cells only in the apical NBL/subventricular zone, the site of retinal mitosis (Alexiades and Cepko 1996; Barton and Levine 2008; Young 1985a), where approximately 10% colocalized with BrdU cells on GD16.5 and GD18.5 (Figure 4A). Although the spatiotemporal pattern of PH3 labeling was similar in control and GLE retinas, GLE significantly increased the number of PH3-IR cells by 25% from GD16.5 to PND3 (Figure 4C).

To explore possible GLE-induced alterations in cell cycle progression, we calculated PH3:BrdU ratios for controls and GLE during the period of significantly increased RPC proliferation. The ratios (0.10–0.12), similar to those obtained with PND1 mouse retinas by [^3^H]-thymidine pulse-labeling (Young 1985a), were not significantly different at any age. This indicates that GLE did not alter the relative time spent in S and M phases.

To further confirm the pulse-labeling BrdU proliferation results, we double-labeled dissociated single cells from developing retinas with an anti-Ki67 antibody and the nuclear stain DRAQ5. Figure 4D reveals that 43.6 ± 0.9%, 23.0 ± 1.4%, and 10.5 ± 1.5% of the control retinal cells were proliferating at PND2, PND4, and PND6, respectively, consistent with rodent studies using different techniques to estimate the percentage of proliferating cells (Alexiades and Cepko 1996; Barton and Levine 2008; Young 1985a). In GLE retinas, the number of proliferating cells significantly increased on PND2, PND4, and PND6 in GLE by 60.5 ± 2.9%, 32.1 ± 2.7%, and 20.9 ± 1.0%, respectively (Figure 4D). These results, which are consistent with the BrdU data (Figure 4B), demonstrate that GLE increased the proliferation of late-born retinal cells.

### GLE did not alter retinal apoptosis during development

**Figure 5:**
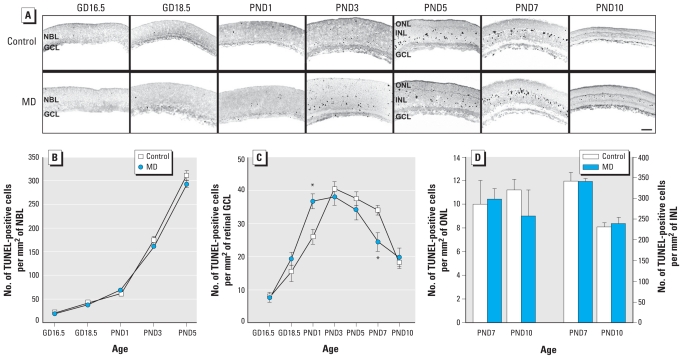
MD GLE does not change the amount or pattern of retinal apoptosis during development. (*A*) TUNEL-positive cells in control and GLE retinas from GD16.5 to PND10. Bar = 100 μm. (*B*) The number of TUNEL-positive cells increased rapidly in the NBL during development, however, there were no age-dependent differences between control and GLE mice. (*C*) Although the kinetics of TUNEL-positive cells differed slightly in the retinal GCL of control and GLE retinas, there was no difference in the number of retinal ganglion cells at PND60 (Figures 1A, 2A). (*D*) There were no differences in the number of TUNEL-positive cells in the ONL or INL between groups. In *B–D*, values are mean ± SE from four to seven retinas at each age per treatment group, with each retina from a different mouse. *p < 0.05 compared with control.

A GLE-induced decrease in apoptosis could increase the number of proliferating RPCs and differentiated neurons. In the NBL of control and MD GLE retinas, TUNEL-positive cells exponentially and similarly increased from GD16.5 to PND5 (Figure 5A,B). In the GCL of control and MD GLE retinas, TUNEL-positive cells increased linearly from GD16.5 to PND3 and then decreased, resulting in an inverted U-shaped curve (Figure 5C). On PND1 and PND7, TUNEL-positive cells in the GCL were slightly, but significantly, increased and decreased in MD GLE mice, respectively. However, we found no significant differences in GCL cellularity at PND60 (Figures 1A and 2A). In the ONL of control and GLE retinas, TUNEL-positive cells were low on PND7 and PND10 and not significantly different. Although numbers of TUNEL-positive cells were 20- to 30-times higher in control and GLE INL than in the ONL on PND7 and PND10, there were no significant differences between treatment groups (Figure 5D). The spatiotemporal patterns and amount of apoptosis in controls were similar to previously published results (He et al. 2003; Portera-Cailliau et al. 1994; Young 1984). Thus, the GLE-induced increase in proliferating RPCs and late-born neurons was not due to decreased apoptosis.

## Discussion

We obtained three novel results with our murine model of GLE. First, GLE produced selective nonmonotonic increases, characterized by an inverted U-shaped dose–response curve, in the numbers of rods and BCs in the adult retina. The increase in rods and BCs did not alter retinal lamination or the proportion of rod and cone BCs. Second, GLE increased and prolonged RPC proliferation without changing the relative S- or M-phase length, initiation of neurogenesis, or pattern of neurogenesis and gliogenesis. Third, GLE did not significantly alter apoptosis or produce reactive gliosis during retinal development and maturation.

The molecular mechanisms responsible for the novel GLE-induced retinal phenotype are unknown. However, the increased and prolonged RPC proliferation accompanied by the selective increase in late-born neurons suggests that two different spatiotemporal components are involved. We deduce that the first involves a modest increase in the number of cell cycles, and the second involves factors that regulate rod and BC fate. Multiple extrinsic and intrinsic factors regulate RPC proliferation and cell fate decisions during development (Livesey and Cepko 2001; Martins and Pearson 2008). Because there are no similar GLE studies, we suggest three logical non-mutually exclusive possible mechanisms that could produce the adult GLE phenotype. First, GLE could enhance the progression of the RPC cell cycle mediated by glutamate and ATP (Martins and Pearson 2008). For example, the pharmacologic antagonism of glutamatergic α-amino-3-hydroxy-5-methyl-4-isoxazolepropionic acid (AMPA)/kainate and *N*-methyl-d-aspartic acid (NMDA) receptors increases RPC proliferation in developing mouse retinas (Martins and Pearson 2008). The noncompetitive inhibition of receptors for NMDA and kainate observed with 1–5 μM added lead (Alkondon et al. 1990; Musshoff et al. 1995) could increase RPC proliferation. Second, GLE might decrease nitric oxide synthase activity in the developing retina, which would increase RPC proliferation. This idea is consistent with findings that nitric oxide regulates RPC proliferation in chick embryo cells (Magalhães et al. 2006) and that moderate-level GLE and lactational lead exposure decreased neuronal nitric oxide synthase in rat brain (Chetty et al. 2001). Third, GLE could selectively increase a subpopulation of RPCs that share a late-born neuronal cell fate, because approximately 20% of all multiple cell clones in rat RPC lineage-tracing studies contained rods and BCs (Turner and Cepko 1987). Thus, this novel GLE model could help uncover the pathways that establish early- and late-born RPCs and specify neuronal versus MGC fate.

## Conclusion

Our results show that GLE in mice with [BPb] at the current low level of concern (CDC 1991) produced persistent retinal alterations characterized by proportional increases in rod and rod BC neurogenesis that likely underlie the observed scotopic ERG supernormality (Fox et al. 2008; Lilienthal et al. 1994; Nagpal and Brodie 2009; Rothenberg et al. 2002). In concert with our previous work (Fox and Boyes 2008), these findings stress the importance of examining developmental-stage–dependent and dose-dependent effects in human and animal toxicology studies. The nonmonotonic effects suggest that HD GLE also triggered rod apoptosis similar to that observed in mice and rats with postnatal-only lead exposure (Fox et al. 1997; He et al. 2003). These latter results are consistent with findings of decreased proliferation and/or neurogenesis in rat hippocampus after GLE and lactational lead exposure with peak [BPb] > 40 μg/dL (Gilbert et al. 2005; Verina et al. 2007). Finally, as suggested by other retinal studies (Tan et al. 2001), the increased number of rods in GLE mice may accelerate age-related retinal degeneration. Our findings raise complex issues for neurotoxicologists, pediatricians, public health regulators, and risk assessors.

## Figures and Tables

**Table 1:** Cell-specific primary antibodies and dyes.

Name	Structure labeled or targeted	Host	Product number and source	Dilution
BrdU	S-phase marker	Rat	Ab6326; Abcam Inc., Cambridge, MA	1:40
ChAT	Amacrine cells	Rabbit	Ab143; Chemicon, Temecula, CA	1:100
Chx10	BCs	Sheep	X1180P; Exalpha Biologicals Inc., Shirley, MA	1:50
Cyclin D3	MGC nucleus	Mouse	MCA1866; Serotec, Raleigh, NC	1:40
DAPI	Nucleus		H-1200; Vector Laboratories, Burlingame, CA	1.5 μg/mL
DRAQ5	Nucleus		BOS-889-001; Alexis Biochemicals; Enzo Life Sciences International Inc., Plymouth Meeting, PA	20 μM
Glutamine synthetase	MGCs	Rabbit	G2781; Sigma Aldrich, St. Louis, MO	1:5,000
Glyceraldehyde 3-phosphate dehydrogenase	Protein loading control	Mouse	MAB 374; Millipore, Billerica, MA	1:300
Ki67	Progenitor cell marker	Mouse	500609; BD, Franklin Lakes, NJ	1:100
Opsins (middle- and short-wavelength sensitive)	Cones	Rabbit	Gifts from C. Craft, University of Southern California, Los Angeles, CA	1:1,000
PH3	M-phase marker	Rabbit	56-0701; Upstate Biotech, Lake Placid, NY	1:40
PKCα	Rod BCs	Rabbit	P4334; Sigma Aldrich	1:1,000
Rhodopsin	Rod photoreceptors	Mouse	MAB5356; Chemicon	1:5,000
